# Monitoring cone-beam CT radiation dose levels in a University Hospital

**DOI:** 10.1259/dmfr.20220213

**Published:** 2023-02-14

**Authors:** Danieli Moura Brasil, Karen Merken, Joke Binst, Hilde Bosmans, Francisco Haiter-Neto, Reinhilde Jacobs

**Affiliations:** 1 Department of Oral Diagnosis, Division of Oral Radiology, Piracicaba Dental School, University of Campinas, Piracicaba, SP, Brazil; 2 OMFS-IMPATH Research Group, Department of Imaging & Pathology, Faculty of Medicine, KU Leuven and Department of Oral and Maxillofacial Surgery, Campus Sint Rafaël, University Hospitals Leuven, Leuven, Belgium; 3 Department of Imaging and Pathology, KU Leuven, Division of Medical Physics & Quality Assessment, Leuven, Belgium; 4 Department of Radiology, UZ Leuven, Leuven, Belgium; 5 Department of Dental Medicine, Karolinska Institutet, Stockholm, Sweden

**Keywords:** dental cone-beam CT, radiation dosimetry, radiation protection, overview clinical workflow

## Abstract

**Objective:**

To present patient dose levels for different CBCT scanners, acquired by a dose monitoring tool in a University Hospital, as a function of field of view (FOV), operation mode, and patient age.

**Methods:**

An integrated dose monitoring tool was used to collect radiation exposure data [type of CBCT unit, dose-area product (DAP), FOV size, and operation mode] and patient demographic information (age, referral department) performed on a 3D Accuitomo 170 and a Newtom VGI EVO unit. Effective dose conversion factors were calculated and implemented into the dose monitoring system. For each CBCT unit, the frequency of examinations, clinical indications, and effective dose levels were obtained for different age and FOV groups, and operation modes.

**Results:**

A total of 5163 CBCT examinations were analyzed. Surgical planning and follow-up were the most frequent clinical indications. For the standard operation mode, effective doses ranged from 35.1 to 300 µSv and 9.26-117 µSv using 3D Accuitomo 170 and Newtom VGI EVO, respectively. In general, effective doses decreased with increasing age and FOV size reduction.

**Conclusions:**

Effective dose levels varied notably between systems and operation modes.Operation mode selection and FOV size were indication-oriented, with larger FOV sizes election serving surgical planning and follow-up. Seeing the influence of FOV size on effective dose levels, manufacturers could be advised to move toward patient-specific collimation and dynamic FOV selection. Systematically monitoring patient doses could be recommended for steering future CBCT optimization.

## Introduction

In most countries, dental exposures are responsible for an important contribution to the total number of medical exposures. More specifically in Belgium, dental exposures account for approximately 25% of all medical exposures.^1^ Dose monitoring is a strategy to track the dose delivered to patients during medical diagnostic imaging acquisitions. DOSE (Qaelum NV, Leuven, Belgium) is a dose management software tool that automatically monitors and reports patient demographic data and radiation exposure data from the image DICOM header information. Based on implemented effective dose conversion factors, DOSE also provides patient effective dose levels. According to the data collected with the dose monitoring system DOSE, around a third of all extraoral dental examinations in the University Hospital Leuven (UZ Leuven, Belgium) are cone-beam computed tomography (CBCT) acquisitions. The considerable share of CBCT imaging in dental exposures is not surprising. Over the past decade, CBCT imaging has been demonstrated to allow for adequate diagnosis and/or elaborate presurgical planning, thereby overcoming problems such as superposition and distortion. As a consequence, it has become a widely accepted tool for various clinical indications related to endodontics, implant dentistry, oral and maxillofacial surgery, trauma diagnosis, and treatment.^2–6^

In terms of dose, the contribution of common 2D dental exposures to the annual population dose is rather limited. CBCT however is less frequentlyused, yetis also associated with increased dose levels that may widely vary between dose levels in the range of panoramic radiography and dose levels equalling medical CT doses.^7,8^The CBCT lifetime attributable cancer risk varies between 2.7 per million for patients older than 60 years and 9.8 per million for children between 8 and 11 years with an average of 6 per million.^9^ In addition, for high-risk groups such as pediatric cleft palate patients, the lifetime radiation exposure is 3 to 5 times higher than non-cleft patients.^10^ Therefore, CBCT exposures should, like all other medical exposures, be clinically justified for each patient based on the principle of keeping radiation dose “As Low as Diagnostically Acceptable being Indication-oriented and Patient-specific (ALADAIP)”.^11^ALADAIP implies that the exposure protocol should not only be based on patient-specific characteristics yet also on indication-oriented requirements to obtain diagnostically acceptable images by the use of the lowest dose possible.^12^ An additional reason for raising concerns about radiation protection of dental CBCT imaging is the wide range of reported doses between different CBCT models.^7,8,13–15^ This wide dose range suggests that optimization of exposures in dental CBCT imaging is still in a preliminary stage and needs to be addressed.

There are many factors affecting radiation dose, with some being patient-related and most being scanner-related. Currently, more than 280 CBCT models are available on the market with different imaging protocols leading to a wide range in performance in terms of both radiation exposure and clinical diagnostic performance.^3,5,16^ Differences among systems are for example selectable FOV range, tube current, tube voltage range, X-ray tube filtration, exposure time, number of projections per rotation, rotation trajectory (360°/180°/partial rotation), focal spot to rotation axis distance, focal spot to detector distance, as well as the presence of dose reduction features like tube current modulation (TCM).

Apart from scanner-related factors, the radiation dose is also influenced by patient-dependent factors, such as age and gender. Organ doses also depend on the fraction of the organ directly exposed to the primary radiation field. For a specific FOV, the larger the organ fraction, the higher the dose. For children, due to their smaller physical size, the organ fraction, and consequently also the organ dose, will be larger compared to adults.^15,17^ Additionally, in the case of CBCT examinations, the primary beam will be closer in height to their radiosensitive organs (*e.g.,* brain and thyroid), resulting in higher exposure.^15^ Furthermore, while organ sizes of children show a rapid increase with age, the variability in adult organ sizes is rather limited.^15^ Therefore, radiation doses should be reported for specific age groups.^8,13,14,18,19^ Another patient-dependent factor is the clinical indication as this determines which FOV size should be selected. Presurgical planning will often demand a larger FOV, thus exposing a larger organ fraction or more radiosensitive organs, with consequently higher patient dose levels.^7^

The aim of this study is to present an overview of patient dose levels, acquired by a dose monitoring tool in a University Hospital and, for the reasons cited above, differentiate these effective doses to the type of CBCT device, operation mode, FOV selection, and patient age, as such to steer future CBCT optimization.

## Methods and materials

### Data collection and analysis

#### Data collection

From the institutional review board, this study on dose monitoring data was exempted from ethical review as the retrospective data used in this study could be regarded as fully anonymous (no patient identification, no clinical or image data access). All CBCT dosimetric data, acquired from January to December 2019 in the Dentomaxillofacial Imaging Center of the University Hospital of Leuven (Leuven, Belgium) were collected using the dose monitoring system DOSE (Qaelum NV, Belgium). This time period was chosen as it was the last year providing patient data from a normal clinical workflow (before the Covid-19 pandemic). Only data of the two most frequently used CBCT systems available in this institution were acquired, *i.e.,* 3D Accuitomo 170 (Morita, Kyoto, Japan), and Newtom VGI EVO (Cefla, Imola, Italy). DOSE provided radiation exposure data and patient demographic information extracted from the patient image DICOM header.^20^ Duplicate examinations and those identified as non-routine acquisitions were eliminated. For each CBCT acquisition, the following scanning and clinical parameters were collected: type of scanner, used operation mode, X-ray tube exposure parameters (tube current in mA, tube voltage in kV, and tube current-exposure time product in mAs), selected FOV (cm²), recorded DAP (in dGy.cm²), patient age, and referral department. Data of 196 and 4,967 acquisitions were respectively recorded for the 3D Accuitomo 170 and the Newtom VGI EVO system, totalizing 5163 CBCT examinations.

#### Descriptive data analysis

Table 1 shows an overview of the recorded acquisition settings for all the examinations performed in our Dentomaxillofacial Imaging Center for both systems. For the Newtom VGI EVO, average recorded DAP values per FOV are provided, as the system applies TCM. In addition, both the recorded DAP values from the patient DICOM header as well as these DAP values corrected with an experimentally determined correction factor are shown. Within this institution, the correctness of the recorded DICOM header DAP values was verified during annual quality control (QC) tests performed by medical physics experts using a VacuDAP meter (VacuTec, Germany). The applied DAP correction factors were based on QC measurements from the past two years. DAP measurements were performed annually on each system for a small, medium, and large FOV for the standard clinical acquisition protocol (three measurements). A correction factor was then established as the average of the ratio between the measured and recorded DAP values. For the 3D Accuitomo 170 system, a correction factor of 0.76 was established. Deviations from the average correction factor were less than 5%. For the Newtom VGI EVO, there were no significant deviations observed between the displayed DAP values on the system and the QC measurements. In the remainder of the study, only the corrected DICOM DAP values were used.

**Table 1.** Clinical acquisition settings for the 3D Accuitomo 170 and Newtom VGI EVO system

CBCT unit	Operation mode	Tube current modulation	Total exposure [mAs]	Tube voltage [kV]	FOV [cm²]	DICOM DAP [dGy.cm²]	Corrected DICOM DAP [dGy.cm²]
3D Accuitomo 170	Standard	No	87.5	90	4 × 4 6 × 6 10 × 5 8 × 8 10 × 10 14 × 10 17 × 12	4.02 8.23 10.0 13.1 18.2 21.7 25.6	3.06 6.25 7.60 9.96 13.8 16.5 19.5
	High-Fidelity	No	154	90	14 × 10 17 × 12	38.1 45.0	29.0 34.2
Newtom VGI EVO	Regular scan	Yes	13–33	110	5 × 5 8 × 5 10 × 5 8 × 8 12 × 8 10 × 10 15 × 12 16 × 16 24 × 19	1.23 1.83 2.12 2.23 3.22 3.31 4.74 6.16 9.54	1.23 1.83 2.12 2.23 3.22 3.31 4.74 6.16 9.54
	High-Resolution	Yes	30–99	110	5 × 5 8 × 5 10 × 5 8 × 8 12 × 8 10 × 10	4.20 6.77 7.82 8.29 11.3 12.5	4.20 6.77 7.82 8.29 11.3 12.5

FOV, field of view; DAP, dose-area product.

For each system, examinations were divided into groups based on the patient’s age, selected FOV, and operation mode. Operation modes used in clinical practice were CBCT-dependent. Due to the similar role that both high-fidelity and high-resolution operation modes play in radiation dose and image quality, they are going to be treated as high-resolution (HR) modes. As well as, both standard and regular scan modes are going to be treated as standard modes.The respective age groups (4–6 year old, 7–11 year old, 12–14 year old, and >= 15 year old) were adopted from DOSE, allowing to provide effective dose conversion factors for these specific age groups.^21^ Finally, the selected FOV (diameter×height in cm^2^) for each CBCT examination was classified into one of the following categories: small (≤40 cm^2^), medium (>40 cm and ≤100 cm^2^), or large (>100 cm^2^).^8^

The diagnostic task and the specific scanned anatomical region can determine, drive, or explain radiation doses (Stratis et al., 2017),^22^ and could therefore be surveyed as well. However, currently, it is not possible to extract this information directly from the DICOM header information.Therefore, the referral department was used for the classification of each examination into the following main clinical indications: endodontics, orthodontic planning, pedodontics, implant placement, surgical planning and follow-up, second opinion radiodiagnosis, and medically compromised patient care. It is important to note that besides the clinical indications directly linked to the different dental specialties, there are two other categories with a broader diagnostic question: second opinion radiologic diagnosis as well as medically compromised patient care. Overviews of the clinical indications and their frequency of imaging requests were established for the different age and FOV groups, as well as the different operation modes on both CBCT systems.

### Effective dose determination

#### Conversion factors

In order to determine patient-effective dose levels, dose conversion factors (CF) were calculated and implemented in DOSE. The implemented CFs were based on the effective dose CFs determined by Stratis et al. (2019),^13^ which were discriminated towards different dental CBCT systems, clinical indications, and patient age (5–15 year-old). These CFs had been obtained via detailed Monte Carlo simulations using anthropomorphic head voxel models.^13,23,24^ The clinical indications referred to the specific anatomical region scanned, *e.g*. single tooth, upper/lower jaw, TMJ, sinuses, and skull.^13^ The 3D Accuitomo 170 and the Newtom VGi EVO were two of the simulated systems. The focus of the work was on pediatric dosimetry, and CFs up to the age of 15 years were determined. However, it can be assumed that the CFs for a 15 year old will be a good estimate for the CFs of an adult as the circumference of the head will not significantly grow after that age.^25^ The CFs were expressed in effective dose (µSv) per total exposure (mAs).^13^

In DOSE, CFs can be implemented for different age groups, and systems, but not for specific clinical indications since this information is not available in the patient DICOM header. CFs are expressed in effective dose (µSv) per DAP (dGy.cm²). Hence, a conversion of the existing CFs was necessary. For both systems, the first step consisted of linking the clinical indications specified by Stratis et al. (2019)^13^ back to available FOVs, for each of the four age groups. The selected FOV option is related to the clinical indication and is available from the patient DICOM header. For each FOV and for each age group, one CF (µSv/mAs) averaged over all possible clinical indications, and ages within that age group could then be determined. In the next step, these averaged CFs were used to determine patient effective doses (µSv) based on patient age, the selected FOV, and the applied mAs. For the 3D Accuitomo 170 system, the effective dose for each available FOV and age group could be determined solely from the program settings available on the system. The exposure parameters are fixed for each patient depending on the selected system setting. For the Newtom VGi EVO, due to the application of TCM, exposure parameters are patient-dependent and patient data were necessary. Therefore, in order of increasing age, data of 28, 143, 306, and 334 patients were collected for each of the four age groups using DOSE. The resulting effective dose (µSv) was divided by the corresponding corrected DICOM DAP value (dGy.cm²). Finally, one average effective dose CF (µSv/dGy.cm²) was obtained for each age group and system, implemented in DOSE (Table 2).

**Table 2.** Effective dose conversion factors (µSv/dGy.cm²) for CBCT devices per age group

CBCT system	4–6 year old	7–11 year old	12–14 year old	>=15 year old
3D Accuitomo 170	16.7	11.7	8.58	7.55
NewtomVGI EVO	19.2	12.9	9.15	7.96

a The conversion factors for each age group are equal for all FOVs.

In addition, the relative error between the effective dose estimated from the newly determined average CFs (µSv/dGy.cm²) and the original CFs (µSv/mAs) was determined. For the Newtom VGi EVO system, mean deviations for the four age groups were lying between 2.3 and 10%. Deviations could be larger than 20%. However, from young to old, 84, 47, 76, and 92% of the deviations were within 20%. For the 3D Accuitomo 170 system, mean deviations were lying between 0.55 and 4.4%. For the four age groups in order of increasing age, 85, 84, 90, and 85% of the deviations were within 20%. This indicated that, although clinical indication and age-specific CFs are preferred, the averaged CFs are still able to provide reliable average dose estimates with acceptable errors for the majority of the patient population.

#### Effective dose calculation

DOSE calculated the effective dose for each examination as follows:

$$E={CF}_{E}x({DAP}_{corrected})$$

with *E* the effective dose in µSv, *CF_E_* the implemented effective dose CF expressed in µSv/dGy.cm², and *DAP_corrected_* the corrected DICOM DAP value in dGy.cm².

**Figure 1.**
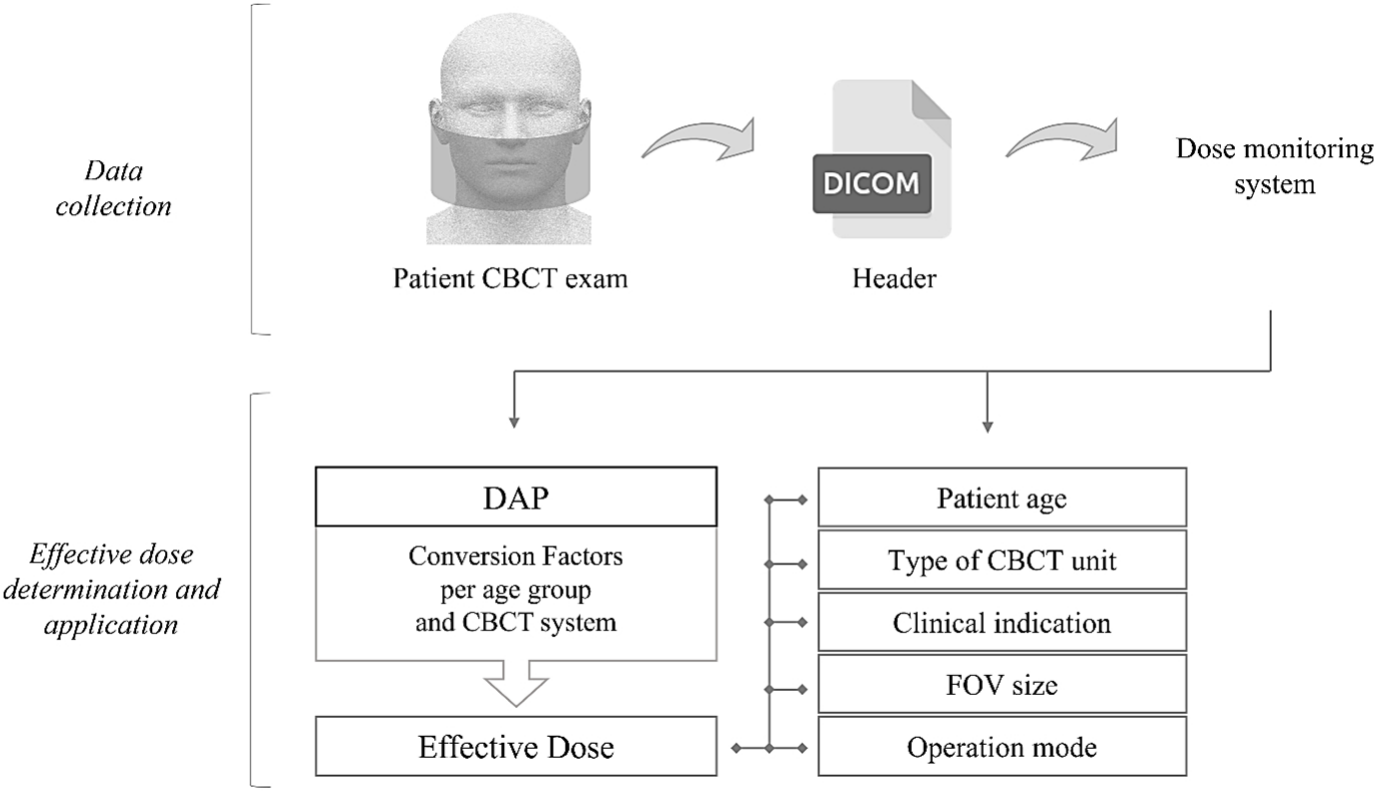
Flowchart of the data collection and effective dose determination and application. After the patient CBCT scan, the acquisition data from the DICOM header was collected by the dose monitoring system. The effective dose values were calculated using the retrieved DAP values as well as conversion factors per age group and CBCT system. Average effective doses were calculated for different categories based on the parameters extracted from the dose monitoring system.

For the Newtom VGI EVO system, the collective effective dose was also determined. It is defined as the sum of all individual patient-effective doses expressed in manSv. Figure 1 summarizes the processes of data collection, effective dose determination, and application using the data collected with the dose monitoring system.

## Results

### Data analysis

**Figure 2.**
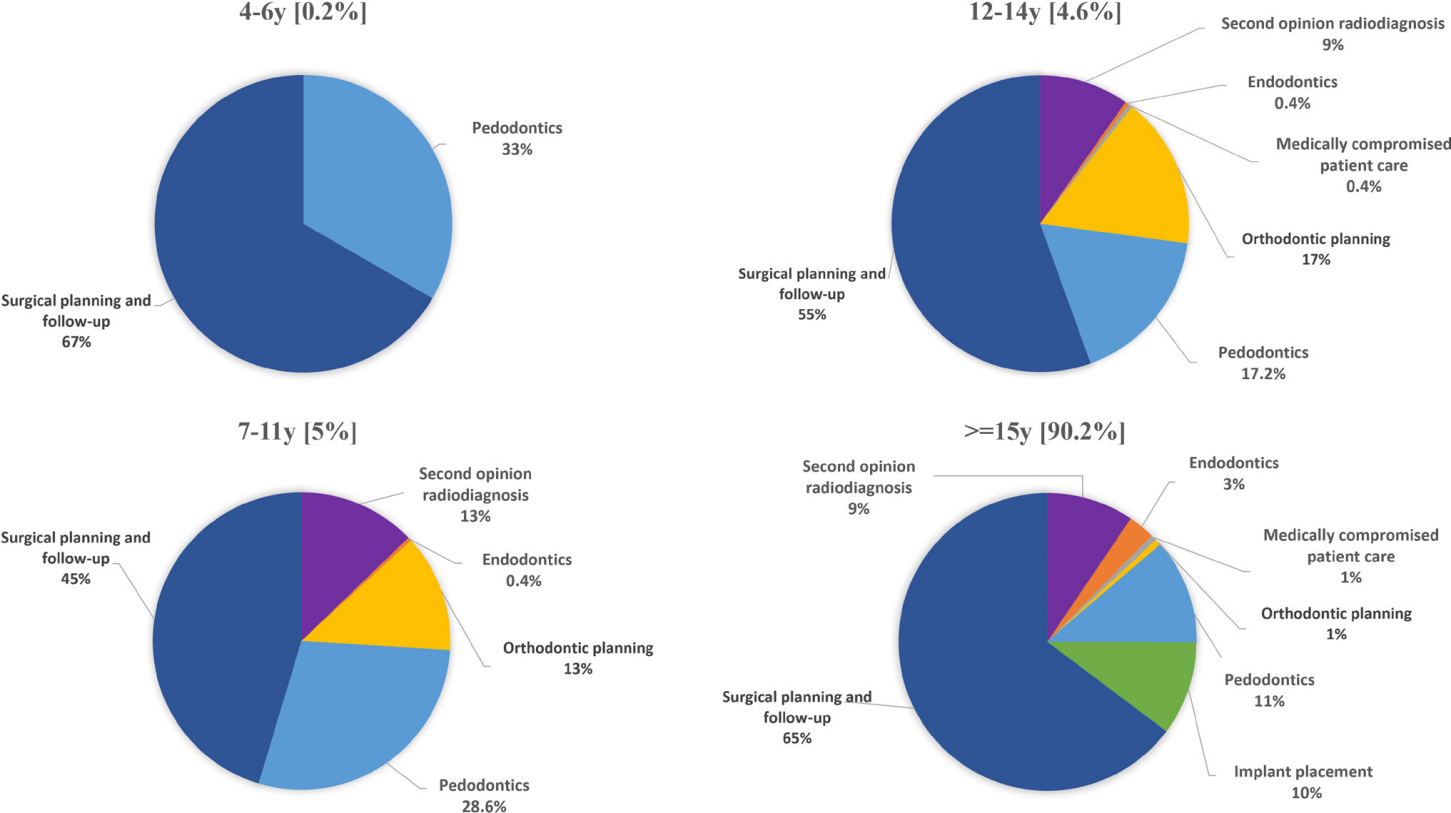
Percentage of imaging requests per clinical indication for each age group, combining the data of both CBCT systems (5163 examinations). y means years old. [%] means the percentage of examinations per each age group.

Figure 2 shows the percentage distribution of imaging requests per clinical indication for each age group and for both CBCT systems combined. For this analysis, no distinction between different operation modes was made. Overall, surgical indications were dominant over radiological diagnostic indications, followed by pedodontics. In children <7 years and in adults, CBCT referral for surgical planning and follow-up even constituted 2/3 of all indications. For <15-year-old age groups orthodontic planning and second opinion radiodiagnosis were the next most common referrals, except for the youngest children group, while for the >= 15-year-old age groups, implant placement and second opinion radiodiagnosis were more common, with only 3% referrals for endodontics. The frequency for other referral categories was not clinically meaningful.

Table 3 shows for each FOV category the percentage of imaging requests for all the clinical indications. No distinction was made between the different operation modes. It can be seen that for all clinical indications, medium FOVs were 2.4 times more requested than large FOVs, while small FOVs were used in only 2.5% of the cases. Moreover, of all referrals, 63% were justified for surgical planning and follow-up using a medium (36%) or large (27%) FOV group. Pedodontics was the second largest group referred for medium FOV imaging (11% of all cases) but constituted the most frequent clinical indication for imaging in the small FOV group (1.5%of all cases).

**Table 3.** Percentage of imaging requests per clinical indication for each FOV group, combining the data of both CBCT systems

FOV size in cm x cm		FOV classification		Frequency [%]	Clinical indication	[%]
3D Accuitomo 170	Newtom VGI EVO					
4 × 4 6 × 6	**5 × 5**	<40 cm^2^	Small	2.5	Pedodontics	1.5
					Implant placement	0.3
					Surgical planning and follow-up	0.2
					Second opinion radiodiagnosis	0.2
					Endodontics	0.2
					Orthodontic planning	0.1
					Medically compromised patient care	0.0
8 × 8, 10 × 5 10 × 10	8 × 5 10 × 5 8 × 8 12 × 8 **10 × 10**	40–100 cm^2^	Medium	69.4	Surgical planning and follow-up	36.0
					Pedodontics	11.0
					Second opinion radiodiagnosis	8.9
					Implant placement	8.9
					Endodontics	2.4
					Orthodontic planning	2.0
					Medically compromised patient care	0.2
14 × 10 17 × 12	15 × 12 16 × 16 **24 × 19**	>100 cm^2^	Large	28.1	Surgical planning and follow-up	27.0
					Second opinion radiodiagnosis	0.5
					Medically compromised patient care	0.4
					Implant placement	0.1
					Pedodontics	0.1
					Endodontics	0.0
					Orthodontic planning	0.0

Bolded data show the most frequently used FOV size within the small, medium, and large FOVs.

Table 4 shows different operation modes in relation to the percentage of imaging requests for all clinical indications. No distinction was made between different FOV sizes. In general, standard operation mode scans were requested 3.7 times more often than HR acquisitions. Again, surgical planning and follow-up were the most frequent referrals, accounting for nearly 60% of the standard acquisitions.

**Table 4.** Percentages of imaging requests per clinical indication per operation mode, combining the data of both CBCT systems

Operation modes	Frequency [%]	Frequency of clinical indications [%]	
Standard		Surgical planning and follow-up	59.8
		Implant placement	7.2
		Second opinion radiodiagnosis	6.5
	78.6	Pedodontics	2.2
		Endodontics	1.4
		Orthodontic planning	0.8
		Medically compromised patient care	0.5
HR		Pedodontics	10.3
		Surgical planning and follow-up	3.5
		Second opinion radiodiagnosis	3.1
	21.4	Implant placement	2.0
		Orthodontic planning	1.3
		Endodontics	1.2
		Medically compromised patient care	0.0

HR, high-resolution.

### Effective dose overview

Table 5 shows the average corrected DICOM DAP and average effective dose for the different age groups and operation modes for each FOV group on both CBCT systems. For the Newtom VGI EVO system, the 95% confidence intervals of the average DAP and effective dose level are shown. In contrast to the 3D Accuitomo 170 system, the average dose values shown for the Newtom VGI EVO system do not only depend on the variability between the doses of the different FOVs within a given FOV group but also on the variability between patients scanned with the same FOV due to the application of TCM. Therefore, the total number of patient examinations used for dose calculations for the Newtom VGI EVO, as well as its percentage over all examinations are also shown in Table 5.

**Table 5.** Average corrected DICOM DAP value and average effective dose level per age group, FOV classification, and operation mode for the 3D Accuitomo 170 and Newtom VGI EVO system. For Newtom VGI EVO system, the 95% confidence interval for the DAP, the 95% confidence interval for the effective dose, and its frequency percentage over all examinations are also presented

Age group	3D Accuitomo 170			NewtomVGI EVO						
	FOV group/ operation mode	Avg corrected DICOM DAP [dGy.cm^2^]	Avg effective dose [µSv]	FOV group/ operation mode	Avg corrected DICOM DAP [dGy.cm^2^]	95% CI DAP	Avg effective dose [µSv]	95% CI effective dose	n	Freq [%]
**4-6y**									**9**	**0.2**
	**Small**			**Small**					**1**	**0.0**
	Standard	4.66	77.7	Standard	-	-	-	-	0	0.0
	HQ	-	-	HQ	3.47	-	66.6	-	1	0.0
	**Medium**			**Medium**					**5**	**0.1**
	Standard	10.5	175	Standard	3.15	-	60.5	-	1	0.0
	HQ	-	-	HQ	4.71	[3.26,6.16]	90.5	[62.7,118]	4	0.1
	**Large**			**Large**					**3**	**0.1**
	Standard	18.0	300	Standard	6.09	[0.768,11.4]	117	[14.7,219]	3	0.1
	HQ	31.6	527	HQ	-	-	-	-	-	-
**7-11y**									**227**	**4.6**
	**Small**			**Small**					**13**	**0.2**
	Standard	4.66	54.5	Standard	0.94	-	12.1	-	1	0.0
	HQ	-	-	HQ	3.78	[3.44,4.11]	48.7	[44.4,53.1]	12	0.2
	**Medium**			**Medium**					**196**	**4.0**
	Standard	10.5	122	Standard	2.12	[1.96,2.29]	27.4	[25.3,29.5]	52	1.0
	HQ	-	-	HQ	6.19	[5.99,6.40]	79.9	[77.3,82.5]	144	3.0
	**Large**			**Large**					**18**	**0.4**
	Standard	18.0	210	Standard	7.51	[6.54,8.49]	97.0	[84.3,110]	18	0.4
	HQ	31.6	369	HQ	-	-	-	-	-	-
**12-14y**									**224**	**4.4**
	**Small**			**Small**					**13**	**0.2**
	Standard	4.66	39.9	Standard	1.04	[0.813,1.27]	9.54	[7.43,11.65]	3	0.0
	HQ	-	-	HQ	3.96	[3.30,4.62]	36.2	[30.2,42.3]	10	0.2
	**Medium**			**Medium**					**156**	**3.1**
	Standard	10.5	89.8	Standard	2.48	[2.34,2.62]	22.7	[21.4,24.0]	81	1.6
	HQ	-	-	HQ	7.14	[6.67,7.60]	65.3	[61.0,69.5]	75	1.5
	**Large**			**Large**					**55**	**1.1**
	Standard	18.0	154	Standard	7.64	[7.10,8.22]	69.9	[64.7,75.1]	55	1.1
	HQ	31.6	271	HQ	-	-	-	-	-	-
**>=15y**									**4507**	**90.8**
	**Small**			**Small**					**58**	**1.2**
	Standard	4.66	35.1	Standard	1.16	[0.460,1.87]	9.26	[3.66,14.9]	3	0.1
	HQ	-	-	HQ	4.72	[4.45,5.00]	37.6	[35.4,39.8]	55	1.1
	**Medium**			**Medium**					**3059**	**61.6**
	Standard	10.5	79.0	Standard	2.93	[2.90,2.96]	23.3	[23.1,23.5]	2263	45.6
	HQ	-	-	HQ	8.59	[8.42,8.77]	68.4	[67.0,69.8]	796	16.0
	**Large**			**Large**					**1390**	**28.0**
	Standard	18.0	136	Standard	7.73	[7.61,7.84]	61.5	[60.6,62.4]	1390	28.0
	HQ	31.6	238	HQ	-	-	-	-	-	-
Total									**4967**	**100%**

y, years old; DAP, dose-area product;CI, confidence interval; avg, average; freq, frequency; HQ, high-quality

For the Newtom VGI EVO system, the number of examinations within the 4–6-year-old age group contributed for only 0.2% to the total amount of CBCT scans taken in the studied period. This resulted in large CIs for the average effective dose estimation. On the other hand, 90.8% of the examinations were performed on patients older than 15 year old. The contribution of the 7–11-year-old and the 12–14-year-old age group to the total amount of examinations was very similar, around 4.5%.

For both systems, it could be observed that within each age group, the average effective dose increased with increasing FOV size for each of the operation modes. In general, a decreasing trend in the effective dose with increasing age could also be observed for a specific acquisition protocol and FOV group.

For the 3D Accuitomo 170 system, effective doses ranged from 35.1 to 300 µSv for the standard operation mode, and from 238 to 527 µSv for the HR mode. For the Newtom VGI EVO system, effective doses ranged from 9.26 to 117 µSv for the standard operation mode, and from 36.2 to 90.5 µSv for the HR mode. For the Newtom VGI EVO system, the collective effective dose was 0.22 manSv.

## Discussion

The present study provided an overview of the CBCT acquisitions and effective dose levels observed in the Dentomaxillofacial Imaging Center of the University Hospital of Leuven,over a period of one year (2019), using the dose monitoring system DOSE. A total of 5163 CBCT examinations were performed in the observed period.

For all age groups, CBCT imaging was requested most frequently for surgical planning and follow-up. CBCT is widely applied for several maxillofacial surgical tasks, playing an important role in applications such asthe evaluation of anatomical structures adjacent to third molars,^26–28^ 3D printing surgical guidance,^29^ virtual reductions of complex jaw fractures,^30^ intraoperative imaging of reconstructive surgery outcome in the region of the midface and the mandible,^31^ radiographic diagnoses and prediction of canine impaction,^32^ tooth segmentation and auto-transplantation planning,^33–35^ and 3D planning for orthognathic surgery.^36,37^ Besides, the second most frequent indication was pedodontics diagnosis and special dental care. The substantial frequency of examinations for these patients emphasizes that radiographers and clinicians should be warned about the importance of using an appropriate scan protocol for specific dental pathological conditions for pediatric examinations.^38^

Each FOV size and operation mode has different applications and should be appropriately chosen according to the structures and anatomical regions to be visualized. Regarding the FOV selection, in this institution, a suitable choice of FOV sizes, guided by the patient’s age and clinical indication, was observed. The medium FOV sizes were requested most often, followed by the large FOVs. This was not surprising as about 2/3 of the indications were surgery-oriented, often requiring anatomical reference data for the virtual patient presurgical planning. It should be noted that, besides the dental care flow, this institution receives a predominant patient flow from maxillofacial surgery for complex maxillofacial procedures related to oncology and congenital deformities.

Regarding the selection of the operation mode, for the Newtom VGI EVO system, the HR mode was more frequently requested than the regular scan mode for small FOVs, mostly aiming to obtain particularized information relevant to teeth and adjacent structures. However, this choice should be avoided when highly dense materials are in the FOV, as in HR operation modes this may result in increased artifacts. For the 3D Accuitomo 170, the HR mode was used only for large FOVs. That was mainly related to patients’ examinations seeking a second opinion diagnosis to exclusion of any problems that could potentially explain pain complaints. In general, for both systems combined, more HR than standard images were requested by the pedodontics and orthodontics departments. For pedodontics, the HR operation mode was about 4.7 times more requested than the standard operation mode. This was not unexpected as this University Hospital is a national reference for special pediatric patients, especially complex cleft palate patients. Although it has been proved that modification of CBCT acquisition parameters like FOV and voxel size does not influence the cleft region assessment, specific diagnostics in children with clefts, *e.g.,* detection of canine eruption patterns or dental anomalies, require an increased resolution.^11^ Moreover, dental anomalies are much more common in patients with orofacial clefts and syndromes, and therefore, these patients should also be carefully evaluated prior to orthodontic treatment.^39^ HR protocols were also used for post-trauma pediatric patients and in case of tooth eruption failure, the latter also being a reason for using HR for orthodontic planning.^40^ In addition, HR protocols were used for surgical planning of impacted canines and high-risk procedures like a close relationship between the third molars and the inferior alveolar canal^41^; as well as to assess complications mostly related to neuro-vascular trauma aiming for the reduction of artifacts that may mask the neurovascular bundle in post-operative implant cases^5^; for cases of therapy-resistant endodontic problems such as root fractures^42,43^ and additional canals in complex anatomy teeth.^44^

It is important to note that the aim of this study was not to provide recommendations for specific clinical indications, but rather an overview of the clinical workflow and the related dose levels. To provide recommendations, another type of study should be conducted, where image quality and radiation dose should be evaluated for a specific diagnostic task and patient group, for different imaging systems and or acquisition settings.

Doses observed in the two studied systems differed notably. Effective doses were for the 3D Accuitomo 170 approximately three times higher when using standard mode than for the Newtom VGI EVO. As expected, the HR modes showed higher dose levels than the standard modes, for both systems. For the 3D Accuitomo 170 system, HR mode effective doses were approximately 1.8 times higher, and for the Newtom VGI EVO system 1.5–4 times. Additionally, for both systems, a decreasing trend in the effective dose with increasing age was observed. This trend was expected as for smaller patients, especially children, organ doses will be higher when exposed to the same acquisition settings.^13,16^ Furthermore, an increasing trend in dose could be observed with increasing FOV size, which was expected from previous studies.^7,45^

For both CBCT systems, average effective dose levels were also estimated for each operation mode based on the effective dose CFs implemented into DOSE. The CFs were system and age-dependent and based on the work of Stratis et al. (2019).^13^ The original CFs by Stratis et al. (2019)^13^ were also determined for specific clinical indications. However, the CFs implemented in DOSE were averaged over all clinical indications as it is not possible to extract this information directly from the DICOM header. Errors on the average CFs were acceptable for the majority of the patient population. Still, in the future, manufacturers could be encouraged to include also the clinical indication in the DICOM header information. This would allow the implementation of indication-specific CFs into the dose monitoring system improving dose estimates even more.

The recorded DAP values were necessary for obtaining the dose estimates. However, it is not uncommon that recorded DAP values on dental X-ray systems are inaccurate. In annual quality control test reports of 10 different dental CBCT systems located throughout Belgium, deviations between 2.5 and 27% from the displayed DAP values have been recorded. Therefore, it should be emphasized here that it is important to verify the correctness of the recorded DAP values on the system, making it possible to use them in dose studies, including optimization. Thereafter, manufacturers should be encouraged to provide accurate DAP values on the systems.

In the past, most dose studies mainly focused on establishing dose estimates for adult patients for a range of CBCT units. Studies focusing on pediatric doses are less common and often did not cover the full pediatric range.^14,18,38^ In the present study, the work by Stratis et al. (2019)^13^ has been used and patient age-specific dose estimates could be provided over the full pediatric range, as well as for adults.

In clinical practice and for surgical applications, the Newtom VGI EVO system was more used considering the larger FOV range and the application of TCM for patient-dose optimization. Yet, indications needing high detail and surely in the presence of high dense objects and/or with a high risk for patient motion were preferably sent to 3D Accuitomo 170. Although the amount of patient data for 3D Accuitomo 170 system is small for different age and FOV groups, this didnot pose problems for dose calculations as the system does not apply TCM: the exposure for a specific operation mode is fixed and does not vary according to the attenuation characteristics of the patient during scanning. Therefore, unlike the NewTom VGI EVO, patient data are not needed for the dose calculations.

Until now, advice and guidelines for various dental and maxillofacial applications concerning dose optimization are limited. Moreover, any existing protocol may not be readily transferable to every CBCT machine, and the manufacturers may not strive to bring the dose to its optimal level.^46^ However, patient dose inspection is an essential tool for quality control, and it is crucial to have well-established and practical methods for dose estimations. The present study used a dose monitoring system to collect radiation exposure data and patient demographic information, and ultimately also patient effective doses. It showed to be a convenient method for providing the most relevant dosimetric data for monitoring, quality assurance, and optimization. Therefore, the collected data could be useful for raising awareness of clinicians regarding the importance of reviewing patient doses. It is important to note, however, that the first reason for an imaging examination is a clinical question that can be either diagnostically or pre-surgically.^47^ The best trade-off between clinical performance and radiation dose should be found: radiation should be minimized while maintaining sufficient diagnostic performance.

## Conclusion

Effective dose levels varied notably between systems and operation modes. Radiation doses showed a decreasing trend with patient age and reduced FOV size. Operation mode and FOV size were indication-dependent. Systematic patient dose monitoring could be recommended to all dental institutions as it might allow for large-scale CBCT optimization studies. Moreover, seeing the impact of the FOV on patient dose, it could be advised to CBCT-developers to move toward patient-specific collimation and dynamic FOV selection, preferably via an automated algorithm to facilitate clinical implementation of the optimal patient-specific and indication-oriented dose.
